# Biophysical Mechanism of the Protective Effect of Blue Honeysuckle (*Lonicera caerulea* L. *var. kamtschatica Sevast.*) Polyphenols Extracts Against Lipid Peroxidation of Erythrocyte and Lipid Membranes

**DOI:** 10.1007/s00232-014-9677-5

**Published:** 2014-05-27

**Authors:** D. Bonarska-Kujawa, H. Pruchnik, S. Cyboran, R. Żyłka, J. Oszmiański, H. Kleszczyńska

**Affiliations:** 1 Department of Physics and Biophysics, Wrocław University of Environmental and Life Sciences, Norwida 25, 50-375 Wrocław, Poland; 2 Department of Fruit, Vegetable and Cereal Technology, Wrocław University of Environmental and Life Sciences, Chełmońskiego 37/41, 51-630 Wrocław, Poland

**Keywords:** Blue honeysuckle polyphenol extracts, HPLC analysis, Antioxidant activity, Erythrocyte membrane anisotropy, Model lipid membranes, Lipid phase transition

## Abstract

The aim of the present research was to determine the effect of blue honeysuckle fruit and leaf extracts components on the physical properties of erythrocyte and lipid membranes and assess their antioxidant properties. The HPLC analysis showed that the extracts are rich in polyphenol anthocyanins in fruits and flavonoids in leaves. The results indicate that both extracts have antioxidant activity and protect the red blood cell membrane against oxidation induced by UVC irradiation and AAPH. The extracts do not induce hemolysis and slightly increase osmotic resistance of erythrocytes. The research showed that extracts components are incorporated mainly in the external part of the erythrocyte membrane, inducing the formation of echinocytes. The values of generalized polarization and fluorescence anisotropy indicate that the extracts polyphenols alter the packing arrangement of the hydrophilic part of the erythrocyte and lipid membranes, without changing the fluidity of the hydrophobic part. The DSC results also show that the extract components do not change the main phase transition temperature of DPPC membrane. Studies of electric parameters of membranes modified by the extracts showed that they slightly stabilize lipid membranes and do not reduce their specific resistance or capacity. Examination of IR spectra indicates small changes in the degree of hydration in the hydrophilic region of liposomes under the action of the extracts. The location of polyphenolic compounds in the hydrophilic part of the membrane seems to constitute a protective shield of the cell against other substances, the reactive forms of oxygen in particular.

## Introduction

Blue honeysuckle (*Lonicera caerulea* L. var. *kamtschatica Sevast.*, Caprifoliaceae), which originates from the Kamchatka peninsula, is known in Asia, particularly in China and Russia, but is little known in America and Europe. Its fruits and leaves are very rich in phenolic acids and flavonoids, which are valuable for human health. In terms of its content of polyphenols, it is similar to the Japanese variety of this plant (*Lonicera japonica*), which is very well known and widely used in Chinese medicine. Extracts from Japanese honeysuckle, because of their antiviral, antimicrobial, antitoxic, antiseptic, and antioxidant properties and cancer treatment support, are used in pharmacological preparations and cosmetics (Kusznierewicz et al. 2011).

Extracts from the leaves and flowers of this plant are used for colds and throat infections. Phenolic components contained in the fruit of honeysuckle berries, i.e., cyanidin and quercetin derivatives, are used in the treatment of diseases of the cardiovascular system and digestive and anticancer therapies (Kong et al. 2003).

It has been reported that phenolic compounds present in blue honeysuckle, such as anthocyanins, chlorogenic acid, quercetin, or kaempferol, are very good scavengers of reactive oxygen and nitrogen, including the hydroxyl radical, and therefore perfectly protect lipids against peroxidation (Rice-Evans et al. 1997). This property of phenolic compounds is very important from the point of view of the protection of human health. Overproduction of the hydroxyl radical in the organism, resulting from toxic factors, with disturbed defensive mechanisms responsible for its removal, leads to oxidation of membrane lipids, whose further consequences are cancer, neurodegeneration, and cardiovascular disease. The effectiveness of plant flavonoids in the removal of free radicals is often greater than the activity of vitamin E or its synthetic counterpart Trolox^®^ (Bonarska-Kujawa et al. 2011b, 2014; Cyboran et al. 2011; Włoch et al. 2013).

A major and very important place of attack by free radicals in the organism is the cell membrane. Oxidation of its components and, in particular, the membrane lipids by free radicals causes structural changes, interfering with membrane functions, which leads to pathological changes in the human organism. The mechanism of the interaction of phenolic compounds with biological membranes, including membrane lipids, has not yet been fully explained. It can be assumed that these substances may bind both electrostatically with the polar groups of membrane phospholipids and hydrophobically with their alkyl chains. The extent of these effects depends on the chemical structure of the compounds. Phenolic compounds, including phenolic acids and anthocyanins, thank to their numerous hydroxyl groups, strongly interact with the hydrophilic part of the membrane, and those of hydrophobic character penetrate deeper into the membrane lipid bilayer, significantly changing its fluidity (Arora et al. 2000).

Studies attest to the protective and medicinal action of different plant polyphenols in the human organism (Chen et al. 2006; Kong et al. 2003; Xiaofei et al. 2011). They protect the body from pathological states and are also effective medications in many diseases. It is assumed that in contrast to conventional medicines, they do not have side effects. Understanding the mechanism of the interaction of plant extracts and their components with the biological membrane at the molecular level will allow us for explaining their positive effects on the human organism. In this connection, a study has been undertaken aimed at understanding the effects of extracts from the leaves and fruit of honeysuckle berry on the structure of the biological and lipid membrane.

In our studies, erythrocytes were treated as a model of the cell, and their membrane as a model of the biological membrane. In addition, the erythrocytes by virtue of their function in the organism are particularly exposed to reactive oxygen species (Lifen et al. 2004; Chaudhuri et al. 2007; Arbos et al. 2008). In the lipid membrane part of the study, liposomes were created from synthetic lipids (DPPC), egg lecithin (EPC), and lipids extracted from the membranes of erythrocytes (red blood cell lipids, RBCL) as well as black membrane lipids (BLM) created from RBCL and EPC. The use of different models of lipid membranes enabled us to determine the effect of extracts on the lipid phase of biological membranes. Microscopic methods were used in the study as well as fluorimetric, electric, calorimetric, and spectrophotometric methods, including Fourier transform infrared (FTIR).

The study had two main objectives. The first was to determine, using the fluorimetric method, the antioxidant activity of extracts from blue honeysuckle leaves (BHL) and fruit (BHF) in relation to red blood cells, in the presence of two oxidation inducers, and hemolytic toxicity of the extracts by means of the spectrophotometric method. The second objective was to determine the physical properties of lipid membranes and red blood cells treated with BHL and BHF, on the basis of the order parameter of the hydrophilic phase, fluidity of the membranes, shape changes of erythrocytes, phase transition temperature of lipid membranes, electric capacity and the degree of hydration of the lipid membranes. Investigations included in this work are concerned with both the antioxidant activity of honeysuckle extracts and their effect on the properties of lipid and biological membranes, taking into account their possible side effects with respect to biological structures. With regard to the extracts from the leaves and fruit of blue honeysuckle, this type of study has not been carried out previously.

## Materials and Methods

### Materials

The fluorescent probes 6-dodecanoyl-2-dimethylaminonaphthalene (Laurdan), 6-propionyl-2-dimethylaminonaphthalene (Prodan), 1,6-diphenyl-1,3,5-hexatriene (DPH), and (1,6-diphenyl-1,3,5-hexatriene) propionic acid (DPH-PA) were purchased from Molecular Probes, Eugene, Oregon, USA. The lipids 1,2-dipalmitoyl-sn-glycero-3-phosphatidylcholine (DPPC), egg yolk lecithin (EPC), and 2,2′-azobis(2-methylpropionamidine) dihydrochloride (AAPH) oxidation inductor were purchased from Sigma Aldrich, Steinheim, Germany. The studies were conducted on isolated pig erythrocyte membranes (RBC), small unilamellar liposomes (SUVs), and multilamellar liposomes (MLVs). Pig erythrocyte membranes were obtained from fresh blood using the method described by Dodge et al. (1963). The content of erythrocyte membranes in the samples was determined on the basis of protein concentration, which was assayed using Bradford’s method (1976), and it was 100 mg/ml. The choice of pig erythrocytes was dictated by the fact that this cell’s percentage of lipids is closest to that of the human erythrocyte, and the blood was easily available. Pig blood was taken each time to a physiological solution of sodium chloride with heparin added.

Small unilamellar liposomes (SUV) were composed of lipids extracted from erythrocyte membranes (RBCL) according to the method described by Maddy et al. (1972), dissolved in a chloroform:methanol solvent and of DPPC. All lipids were evaporated to dryness under nitrogen. Subsequently, a phosphate buffer of pH 7.4 was added, and MLVs were formed by mechanical shaking. Then SUVs were formed using a Sonics VCX750 sonicator (Sonics & Materials, Inc.). Multilamellar liposomes were composed of DPPC or EPC. Lipids were evaporated to dryness under nitrogen, then phosphate buffer was added, and liposomes were formed by mechanical shaking.

Fruit and leaves of the blue honeysuckle (*Lonicera kamtschatica*) variety called “Green” were harvested from the Garden of Medical Plants Herbarium of the Medical University of Wrocław, Poland. Plant extracts were obtained from the Department of Fruit, Vegetable and Cereal Technology, Wroclaw University of Environmental and Life Sciences. Polyphenols were isolated from leaves and fruits by extraction with water containing 200 ppm of SO_2_, the ratio of solvent to leaves or fruits being 3:1. The extract was absorbed on Purolite AP 400 (UK) for further purification. The polyphenols were then eluted out with 80 % ethanol, concentrated, and freeze-dried. The percentage content of polyphenols in the extracts was determined by high-performance and ultra-performance liquid chromatography (HPLC and UPLC). Phenolic compounds were identified with the HPLC/DAD method, and the method of UPLC/ESI/MS analysis described extensively by Oszmiański et al. (2011).

Detailed quantitative and qualitative contents of phenolic compounds in the extracts from leaves and fruits of blue honeysuckle are given in Table 1.

**Table 1:** Percentage content and characterization of phenolic compounds of extracts of blue honeysuckle (*Lonicera kamtschatica*) fruits (BHF) and leaves (BHL) using their spectral characteristics in HPLC–DAD (retention time, *λ*_max_) and positive and negative ions in UPLC–ESI–MS [M-H]^−^

Peak	Phenolic compounds	*R*_t_ (min)	*λ*_max_ (nm)	[M-H]^−^	Content (%)	
					Fruits	Leaves
1	Neochlorogenic acid	7.29	320	353	0.28	0.47^a^
2	Caffeoyl tartaric	8.37	320	311		0.1^a^
3	Chlorogenic acid	10.55	320	353	2.13	6.71^a^
4	Cryptochlorogenic acid	11.04	320	353		0.38^a^
5	Cyanidin-3,5-diglucoside	14.25	515	611	1.15	
6	Cyanidin-3-glucoside	17.00	517	449	20.94	
7	Cyanidin-3-rutinoside	18.07	519	463	1.86	
8	Di-O-caffeoylquinic acid derivatives	18.83	320	515		0.56^a^
9	Quercetin-3-O-rutinoside	19.20	355	609	1.16	1.95^a^
10.	Quercetin-3-O-galactoside	20.11	355	463	0.36	0.65^a^
11	Peonidin-3-glucoside	20.17	519	463	0.69	
12.	Quercetin-3-O-glucoside	20.82	355	549	0.11	9.08^a^
13.	Quercetin-3-O-glucosylxyloside	21.38	354	595		2.09^a^
14.	3,5-Di-O-caffeoylquinic acid	23.3	320	515		7.03^a^
15.	Kaempferol-3-O-glucoside	24.20	346	447	0.05	0.46^a^
16.	Kaempferol-3-O-galactoside	24.46	346	447		0.4^a^
Total					28.72	29.7^a^

^a^Oszmiański et al. (2011)

## Methods

### Investigation of Extract Amphiphilicity

The partition coefficient (*P*) between octanol and phosphate buffer (pH 7.4) for the polyphenolic compounds contained in the extracts was determined by the spectrophotometric method described by Nenadis et al. (2003). Briefly, the partition coefficient *P* was calculated using the formula

1 $$P = \frac{{A_{\text{x}} }}{{A_{\text{o}} - A_{\text{x}} }}$$

where A_o_ = absorbance corresponding to the maximum concentration of the used compounds in the organic phase, represented by octanol, *A*_x_ = absorbance corresponding to the concentration of used substances that remained in the organic phase. The spectra were recorded using a spectrophotometer (Cary 300 Bio, Varian) in the range 200–380 nm (UV).

The partition coefficient of the polyphenol compounds between octanol and phosphate buffer was expressed as log *P*. With increasing negative value of log *P,* the hydrophilic nature of the compounds increases and so does also their affinity to aqueous media.

### Hemolytic Activity of Extracts and Osmotic Resistance of Erythrocytes

The hemolytic and osmotic experiments were conducted on fresh, heparinized pig blood and investigated using the spectrophotometric method described earlier by Cyboran et al. (2012) with minor modification. For washing erythrocytes, and in the experiments, an isotonic phosphate solution (pH 7.4) was used, and the erythrocytes were incubated in the same solution but containing proper amounts of the extracts. After modification, the hemoglobin content was assayed using a UV–Vis spectrophotometer (Cary 300 Bio, Varian) at 540-nm wavelength. The percentage hemoglobin concentration in the supernatant of totally hemolyzed cells was assumed as the measure of the extent of hemolysis.

For osmotic resistance, the erythrocyte cells modified by extracts were taken and suspended in test tubes containing NaCl solutions of 0.5–0.86 % concentration and to an isotonic (0.9 %) NaCl solution. After that the percentage of hemolysis was measured with a spectrophotometer at *λ* = 540 nm. On the basis of obtained results, the relation between the percentage of hemolysis and NaCl concentration in the solution was determined. Next, using the plots obtained, the NaCl percentage concentrations (C_50_) that caused 50 % hemolysis were determined. The C_50_ values were taken as a measure of osmotic resistance.

### Erythrocyte Shapes

For investigation with the optical microscope, the red cells separated from plasma were washed four times in saline solution and suspended in two of the same solutions containing 0.01 and 0.1 mg/ml of BHL and BHF, respectively. After modification (hematocrit 2 %, 1 h, 37 °C), the erythrocytes were fixed with a 0.2 % solution of glutaraldehyde. After that, the red cells were observed under a biological optical microscope (Eclipse E200, Nikon, Tokyo, Japan) equipped with a digital camera. The photographs made it possible to count erythrocytes of various shapes, and then the percentage was found of the two basic forms (echinocytes and stomatocytes) in a population of ca. 800 cells. The individual forms of erythrocyte cells were assigned morphological indices according to the Bessis scale (Bessis 1977), which for stomatocytes assume negative values from −1 to −4 and for echinocytes positive values from 1 to 4.

For investigation with the electron microscope, the erythrocytes, after modification with BHL and BHF at 0.1 mg/ml, were fixed for 48 h in a 2.5 % solution of glutaraldehyde. After that, the preparations were washed in phosphate buffer for 20 min, and then the material was dehydrated in acetone at increasing concentrations (30, 50, 60, 70, 80, 90, and 100 %). Each sample was washed for 15 min in an appropriate concentration, the material remaining in pure acetone for 30 min. Next, the erythrocytes were dried for 12 h at room temperature. Erythrocytes thus prepared were deposited on object stages and subjected to X-ray microanalysis by means of an X-ray analyzer by Bruker (Billerica, MA), AXS Quantax, collaborating with the program ESPRIT ver. 1.8.2. Next, the samples were coated with gold using a Scancoat 6 (Edwards, London) sprinkler. The material’s ultrastructure was analyzed using an EVO LS15 scanning microscope (Zeiss, Oberkochen, Germany) with SE1 detector, under high vacuum and accelerating voltage EHT = 20 kV.

### Fluidity and Packing Arrangement of the Membranes

The effect of BHL and BHF extracts on packing arrangement and fluidity of lipids in the erythrocyte membrane and model lipid membrane (RBCL liposomes) was investigated using the fluorimetric method described earlier by Bonarska-Kujawa et al. (2011a), with minor modification. Fluorescence intensity was measured by using the fluorescent probes Prodan, Laurdan, and DPH. These fluorescent probes were chosen, because they become incorporated in different regions of the lipid bilayer. The active part (fluorophore) of the DPH probe is located in the hydrophobic and that of Prodan and Laurdan in hydrophilic regions of the bilayer (Lakowicz 2006). Such differentiated incorporation of the probes gives an insight into the structural changes caused by incorporation of components of the BHL and BHF extracts.

The control samples contained erythrocyte membrane suspension and a fluorescent probe, while the investigated samples in addition contained appropriate concentrations of the compounds studied. Fluorescence intensity was measured at 37 °C by using the three fluorescent probes Prodan, Laurdan, and DPH, whose concentration in the samples was 10 μM, while concentrations of the extract were within the range 0.005–0.05 mg/ml. The measurements were conducted with a Cary Eclipse fluorimeter (Varian, Palo Alto, CA) equipped with a Peltier DBS temperature controller (temp. accuracy ±0.1 °C). The excitation and emission wavelengths were as follows: for DPH, *λ*_ex_ = 360 nm, and *λ*_em_ = 425 nm. The excitation wavelength for Laurdan and Prodan was 360 nm, and the emitted fluorescence was recorded at 440 and 490 nm.

Small unilamellar liposomes (SUV) were composed of lipids extracted from erythrocyte membranes (RBCL) and were formed using a sonicator in the presence of fluorescent probes. Control samples contained only lipid suspension with fluorescence probes at 1,000:1 of lipids:probe molar ratio, an appropriate compound at 0.005–0.05 mg/ml concentration being added to the remaining samples.

On the basis of the measured fluorescence intensity of probes, the values of fluorescence anisotropy (A) for the DPH probe and generalized polarization (GP) for Laurdan and Prodan were calculated using the formula described by Parasassi et al. (1998).

### Temperature of Phase Transition in Lipid Membranes

In the calorimetric studies, the effect of tested extracts on the pre-transition (*T*_p_) and main-transition (*T*_m_) temperature of DPPC was analyzed. For that purpose, differential scanning calorimetry (DSC) and steady-state fluorescence spectroscopy were used.

Samples for DSC consisted of multilamellar vesicles (MLV) made of DPPC and modified with the BHF and BHL extracts (Tien 1974). The measurements were made with Thermal Analysis System D.S.C. 821^e^ (Mettler-Toledo, LLC, Columbus, OH), 2 °C/min scanning rate. The samples contained multilamellar liposomes formed of DPPC in the presence of BHL and BHF. Small unilamellar liposomes (SUV) with probes were formed by sonication of DPPC dispersion in a buffer for 15 min at 20 kHz. Control samples contained lipid suspension and a suitable fluorescence probe at 1,000:1 molar ratio. An appropriate compound at 0.005–0.05 mg/ml was added to the remaining samples. Fluorescence intensity was measured with the Laurdan, Prodan, and DPH probes. The measurements were made at different temperatures. For liposomes formed from one kind of lipid, the measurements were made above and below the main phase transition.

### Capacitance of Black Lipid Membranes

Monitoring the impact of used extracts on the electrical properties of BLM may contribute to a better understanding of the molecular mechanisms underlying their interaction with the lipid membrane (Fettiplace et al. 1975). Physical parameters describing electric properties of lipid membranes, such as capacity of BLM, depend on the structure of the membranes and the developments in the surrounding environment. The black lipid membrane can be treated as a capacitor, whose capacity (*C*) is described by the following formula (Everitt and Haydon 1968):

2 $$C = \frac{{\varepsilon \,\varepsilon_{0} \,S}}{d}$$

where: *ε*_0_ = electric permittivity of vacuum, *ε* = electric permittivity of membrane, *S* = membrane surface area, *d* = thickness of lipid bilayer (membrane).

BLMs were formed, using the Mueller-Rudin method (Mueller et al. 1962), from lipids extracted from erythrocyte membranes (BLM_E_) and egg phosphatidylcholine (BLM_EPC_). BLMs were formed from a solution of lipids dissolved in n-decane, the lipid concentration being 20 mg/ml. They were formed on a 1.05 mm hole in the partition of a two-compartment chamber filled with a 0.9 % NaCl solution. The formation of the membranes was monitored visually and electrically by measuring the membrane capacitance, using a four-electrode system and the capacitance-to-period conversion method (Kalinowski and Figaszewski 1995). It was assumed that the membrane formation process was completed when the capacitance drift did not exceed 10 pF/min.

Extracts were pipetted into the solution and carefully mixed after a bilayer membrane was spontaneously formed. Extracts at 10 and 50 µg/ml were added on both sides of the lipid membrane after checking its stability (Δ*C* < 10 pF). The electrical capacity C was measured for 70 min after addition of extracts. Measurements were performed at room temperature (23–25 °C) using Ag/AgCl electrodes of 0.5 cm^2^ average area, immersed directly into the electrolyte solutions. Membrane surface area was determined on the basis of membrane photographs recorded in transmitted light.

To determine the effect of the extracts on membrane capacity, the results were expressed as relative change in membrane capacitance, i.e., capacitance of modified membrane (*C*_Mt_) to the membrane capacitance before modification (*C*_Mo_).

### Hydration of Lipid Membrane

The experiments were performed with EPC and DPPC liposomes (MLVs). IR spectra of 70 mM lipid suspension were taken. Liposomes were prepared by the standard shaking method. Extract concentration was 0.05 mg/ml. The preparations were intensively shaken with a VORTEX under nitrogen at room temperature with EPC and at 45 °C with DPPC liposomes. The measurements were performed using a Thermo Nicolet 6700 MCT (Thermo Fisher Scientific, Waltham, MA) with ZnSe crystal at room temperature. Each single spectrum was obtained from 128 records at 2 cm^−1^ resolution in the range 700–4,000 cm^−1^. Preliminary elaboration of a spectrum was done using the EZ OMNIC v 8.0 Program, also by Thermo Nicolet. After filtering the noise out of the extracts spectrum, the spectrum of the buffer solution was removed, and the baseline corrected.

In spectra thus prepared, we examined three bands located in the range 3,000–2,800 cm^−1^ from vibrations of CH_2_ and CH_3_ groups of alkyl chains, in 1,780–1,700 cm^−1^ and 1,300–1,200 cm^−1^, which correspond to carbonyl group (C=O) vibrations, and 1,000–940 cm^−1^ corresponding to choline group (N-CH_3_) vibrations.

The frequency of methylene and methyl groups of alkyl chains depends on mobility (fluidity) of the chains and increases with increasing temperature or during transition from the gel state to the liquid-crystalline state. An increase in the wavenumber of these bands testifies to increased fluidity of the hydrophobic part of membrane. The carbonyl group and even more the phosphate groups form hydrogen bonds with water. The carbonyl group can bind one molecule of water, while the phosphate group can bind a few. Hence the carbonyl and phosphate bands of phospholipids are the sum of the vibrations of C=O or phosphate groups that are at different degrees of hydration (Lewis et al. 1994; Attar et al. 2000). Vibrations of C=O of phosphate groups which do not have water bonds are represented by the wavenumbers ≈1,740 and ≈1,260 cm^−1^. Each bound water molecule moves these values by about 20 cm^−1^ in the direction of smaller values. The changes observed in these bands testify, therefore, to changes in the degree of hydration of the carbonyl and phosphate groups.

### Antioxidant Activity of Extracts

Antioxidant activity of BHF and BHL extracts was determined using the fluorimetric method described previously by Bonarska-Kujawa et al. (2012), with minor modifications. These studies were carried out on erythrocyte membranes. The DPH-PA probe was used in the experiments. Suspensions of erythrocyte membranes were treated with UVC radiation and a chemical oxidation inducer (AAPH) for 30 min. Free radicals, released in the process of UVC irradiation or AAPH decomposition, cause quenching of DPH-PA fluorescence and a decrease in fluorescence intensity. Relative fluorescence, i.e., the ratio of UVC or AAPH-oxidized probe fluorescence to the initial fluorescence of the probe, was adopted as a measure of the extent of lipid oxidation. Excitation and emission wavelengths of DPH-PA probe were *λ*_ex_ = 364 nm and *λ*_em_ = 430 nm.

### Statistical Analysis

Statistical analysis was carried out using Statistica 10.0 (StatSoft Inc., Tulsa, OK). All the experiments were performed at least in triplicate unless otherwise specified. Analysis of variance was carried out, and significance between means was determined using Dunnett’s post hoc test. Results are presented as mean ± SD. Significance levels were defined at *p* = 0.05.

## Results

### Amphiphilicity of the Extracts

The log *P* parameter calculated using formula (1) confirms the hydrophobic character of the compounds, which makes them more prone to the organic phase, represented here by octanol. Negative values of the log *P* parameter indicate hydrophilic character of the compounds and greater affinity to the aqueous phase. The values of the coefficients of amphiphilicity (log *P* ± SD) are for BHL −0.076 ± 0.009, for BHF −0.360 ± 0.023, and for the standard antioxidant Trolox^®^ −0.805 ± 0.044, which indicates the hydrophilic nature of the polyphenolic compounds contained in the BHL and BHF extracts, the fruit extract being the more hydrophilic. The sequence of increasing hydrophobicity is as follows: Trolox < BHF < BHL.

### Hemolytic Activity of Extracts and Osmotic Resistance of Erythrocytes

In the presence of BHL and BHF, in the concentration range from 0.01 to 0.05 mg/ml, there was no increased hemolysis of erythrocytes in relation to the control group of cells. At 0.05 mg/ml concentration of BHF, the hemolysis was close to 4.5 %, and for BHL, it was at the level of 0.5 % (Table 2).

**Table 2:** Averaged percentage of hemolyzed erythrocytes induced with different concentrations of extracts from fruits and leaves of blue honeysuckle

Extract	BHF	BHL
Concentration [mg/ml]	Percent of hemolysis ± SD	
Control	5.10 ± 0.56	5.10 ± 0.56
0.01	1.52 ± 1.07	0.34 ± 0.05
0.02	1.89 ± 0.38	0.38 ± 0.28
0.03	2.84 ± 0.78	0.37 ± 0.16
0.04	3.55 ± 1.00	0.34 ± 0.24
0.05	4.43 ± 1.37	0.41 ± 0.35

In the study of the impact of the extracts on erythrocyte osmotic resistance, no significant differences were found in the degree of hemolysis in the control cells and those modified with the extracts at different concentrations of sodium chloride. The *C*_50_ values obtained for blood cells treated with BHL and BHF of 0.01 mg/ml concentration were as follows: control 0.7385 ± 0.0170 %, BHL 0.7353 ± 0.0331 %, and BHF 0.7242 ± 0.0152 % of NaCl (%) concentration. They point to a slight increase in osmotic resistance of erythrocytes in the case of the BHF extract. The results obtained indicate that the extracts in the used range of concentrations cause only a slight increase in the erythrocyte resistance to changes in osmotic pressure.

### Erythrocyte Shapes

**Fig. 1:**
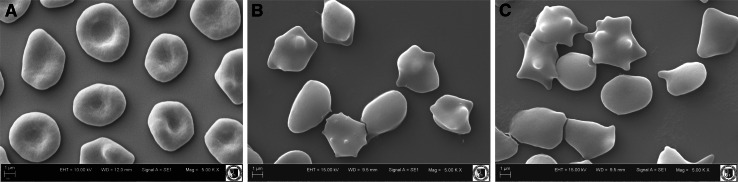
Shapes of unmodified erythrocytes (**a**) and modified with BHL (**b**) and BHF (**c**) observed with the electron microscope, at 0.1 mg/ml concentration

Figure 1a–c shows the erythrocyte shapes as observed in scanning electron microscopy modified with the extracts at 0.1 mg/ml concentration. Table 3 shows the proportions of the various forms of cells in a population of erythrocytes modified with BHL and BHF at 0.1 and 0.01 mg/ml. As seen in the figures, the extracts induce various forms of echinocytes, mainly discoechinocytes. Studies by Deuticke (1968) and Iglic et al. (1998) showed that formation of echinocytes occurs when amphiphilic molecules are incorporated in the outer monolayer of the erythrocyte membrane. It can thus be assumed that the blue honeysuckle extracts concentrate mainly in the outer monolayer of the erythrocyte membrane when inducing echinocytes and practically do not permeate into the inner monolayer of the membrane.

**Table 3:** Mean percentage of erythrocyte shapes formed in the presence of blue honeysuckle leaves and fruits extracts applied at 0.01 and 0.1 mg/ml

Average percent share of individual forms of erythrocytes ± SD					
Extracts		Leaves extract (BHL)		Fruit extract (BHF)	
Shape of erythrocytes (morphological index)	Control	0.01 mg/ml	0.1 mg/ml	0.01 mg/ml	0.1 mg/ml
Spherostomatocytes (−4)	0	0	0	0	0
Stomatocytes II (−3)	0	0	0	0	0
Stomatocytes I (−2)	1.16 ± 0.46	0	0	0	0
Discostomatocytes (−1)	9.07 ± 0.45	0.97 ± 1.01	0.57 ± 0.80	0.95 ± 0.44	0.77 ± 0.56
Discocytes (0)	60.04 ± 0.74	23.98 ± 0.29	22.18 ± 0.32	22.39 ± 0.20	15.52 ± 0.64
Discoechinocytes (1)	19.11 ± 0.57	60.04 ± 0.64	36.52 ± 0.31	51.8 ± 0.28	59.0 ± 0.68
Echinocytes (2)	8.30 ± 0.30	15.01 ± 0.81	35.95 ± 0.27	24.86 ± 0.28	24.71 ± 0.35
Spheroechinocytes (3)	2.32 ± 0.62	0	4.78 ± 0.49	0	0
Spherocytes (4)	0	0	0	0	0

### Fluidity and Packing Arrangement of the Membranes

The DPH steady-state anisotropy is primarily related to the rotational motion restriction due to the hydrocarbon chain packing order. Therefore, the observed decrease of this parameter can be explained by a structural perturbation of the bilayer hydrophobic region due to the incorporation of investigated compounds. The effect of BHL and BHF on fluidity of the lipid phase of erythrocyte membranes and liposomes formed from RBCL was studied on the basis of fluorescence anisotropy (A) measured with the fluorescence probe DPH. Small changes in fluorescence anisotropy were recorded only for erythrocyte membranes affected by the BHF extract (Table 4).

**Table 4:** Values of fluorescence anisotropy of DPH probe and values of generalized polarization (GP) of the Laurdan probe for the erythrocyte membrane and liposomes formed from erythrocyte lipids modified by BHL and BHF extracts at 37 °C

Extract	BHF		BHL	
Membrane	Erythrocyte membranes	Liposomes from erythrocyte lipids	Erythrocyte membranes	Liposomes from erythrocyte lipids
Concentration (mg/ml)	Anisotropy (A) ± SD			
Control	0.245 ± 0.007	0.202 ± 0.002	0.245 ± 0.007	0.202 ± 0002
0.005	0.238 ± 0.007	0.194 ± 0.001	0.233 ± 0.007	0.202 ± 0.004
0.0075	0.238 ± 0.007	0.194 ± 0.001	0.233 ± 0.010	0.200 ± 0.002
0.01	0.236 ± 0.005	0.195 ± 0.001	0.233 ± 0.013	0.200 ± 0.002
0.025	0.235 ± 0.008	0.197 ± 0.001	0.234 ± 0.005	0.198 ± 0.002
0.05	0.234 ± 0.005	0.198 ± 0.002	0.230 ± 0.006	0.200 ± 0.001
Concentration (mg/ml)	Generalized polarization (GP) ± SD			
Control	0.398 ± 0.041	0.280 ± 0.020	0.398 ± 0.041	0.280 ± 0.020
0.005	0.319 ± 0.016	0.268 ± 0.024	0.277 ± 0.029	0.262 ± 0.012
0.0075	0.312 ± 0.012	0.265 ± 0.024	0.256 ± 0.030	0.254 ± 0.012
0.01	0.312 ± 0.032	0.267 ± 0.027	0.227 ± 0.054	0.249 ± 0.015
0.025	0.311 ± 0.014	0.264 ± 0.027	0.203 ± 0.007	0.237 ± 0.021
0.05	0.254 ± 0.013	0.270 ± 0.031	0.166 ± 0.043	0.228 ± 0.020

The results indicate that the extracts do not alter fluidity of the erythrocyte membrane in the region occupied by acyl chains of fatty acids of lipid molecules. No changes were observed for BHL and BHF in the hydrophobic region where the unspecific DPH probe becomes located. It can thus be postulated that the phenolic compounds practically do not concentrate in the hydrophobic lipid phase of the erythrocyte membrane (Suwalsky et al. 2009).

We have also investigated, using the Laurdan probe, the degree of order in the hydrophilic part of the erythrocyte membrane and liposomes formed from RBCL. The calculated values of general polarization (GP) decreased with increasing extract concentration (Table 4), which is indicative of increasing disorder in the hydrophilic part of the lipid layer and the presence of the compounds in that area. Though the changes induced by BHF in the liposome (RBCL) and erythrocyte membranes are smaller than those induced by BHL, the conviction remains that the compounds of the latter extract become incorporated into erythrocyte and liposome membranes and induce greater disorder in the hydrophilic part of membranes. In the presence of BHL, there occurs a concentration-dependent decrease in GP of Laurdan probe. This result indicates that the extract changes the arrangement of the hydrophilic region of the erythrocyte membrane, probably through penetration into this area or absorption on the surface of the membrane.

### Temperature of Phase Transition of Lipid Membranes

Using differential scanning calorimetry (DSC) and fluorimetric measurements, we studied the effect of BHL and BHF on phase transitions and fluidity of DPPC membranes.

**Fig. 2:**
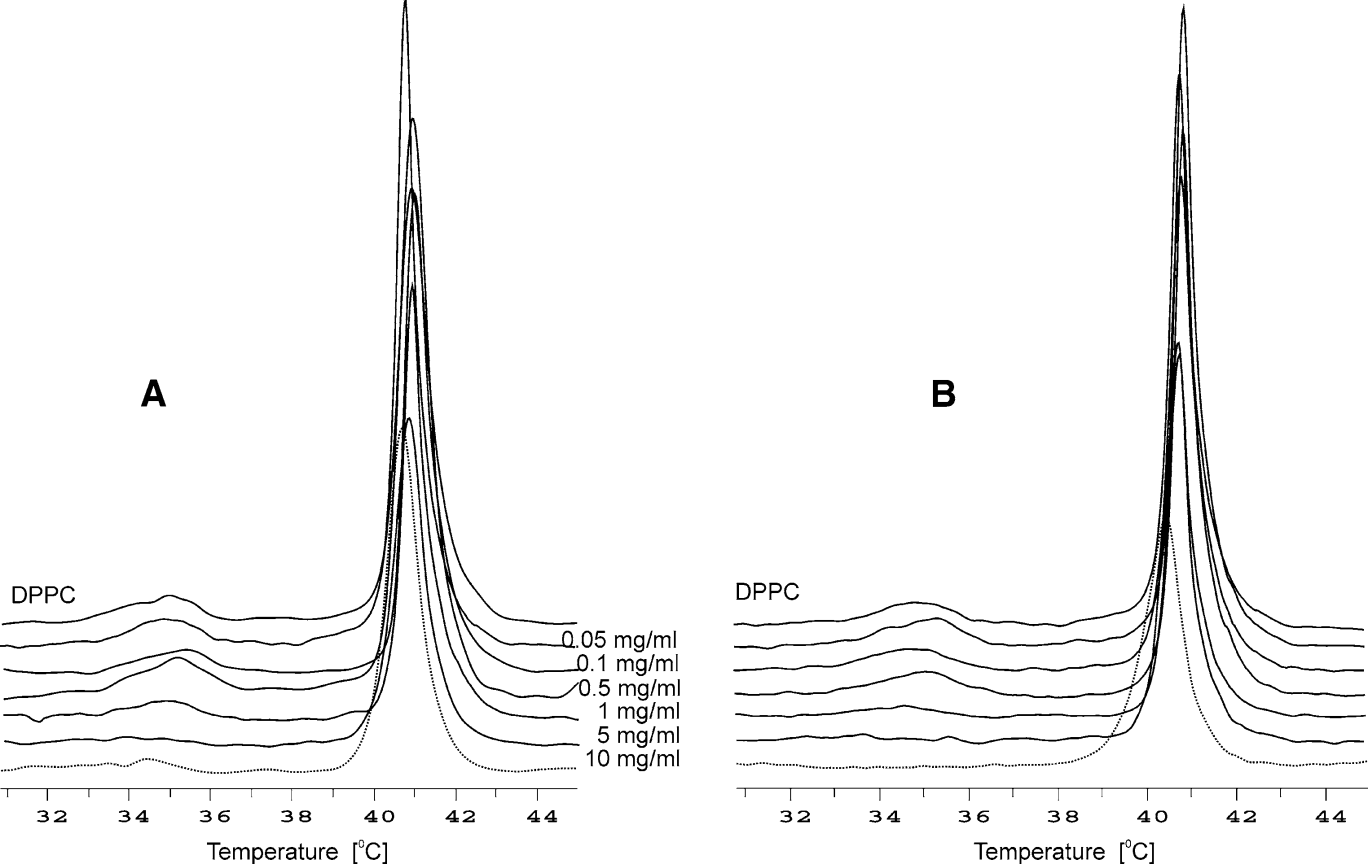
DSC heating curves for MLVs DPPC liposomes containing different concentrations of BHL (**a**) and BHF (**b**). The *curves* are normalized for the amount of DPPC; scan rate 2 °C/min

The calorimetric measurements were made for a number of selected concentrations of the extracts within the range 0.05–10 mg/ml. Even at the highest concentrations of BHL and BHF (10 mg/ml), we did not observe significant changes in temperature of the main phase transition *T*_m_ (control 41.2 ± 0.2 °C, BHL 40.8 ± 0.2 °C, BHF 40.5 ± 0.2 °C), while the half width of the peak (*T*_1/2_) did not change for BHL (control 0.7 ± 0.2 °C, BHL 0.8 ± 0.2 °C) or increased slightly for BHF (control 0.7 ± 0.2 °C, BHF 1.0 ± 0.2 °C) (Fig. 2).

The extracts tested do not change the temperature of the pre-transition, and the main phase transition of a model membrane formed from DPPC, which suggests that extract components do not induce significant changes in fluidity of the bilayer and membrane structure. Increased concentration of the extracts caused only a reduced heat effect originating from the DPPC pre-transition, until it completely disappeared, which may suggest that the tested compounds cause only small changes in the polar part of the membrane. The DSC results suggest that extracts do not change the structure and organization of the phospholipid bilayer.

**Fig. 3:**
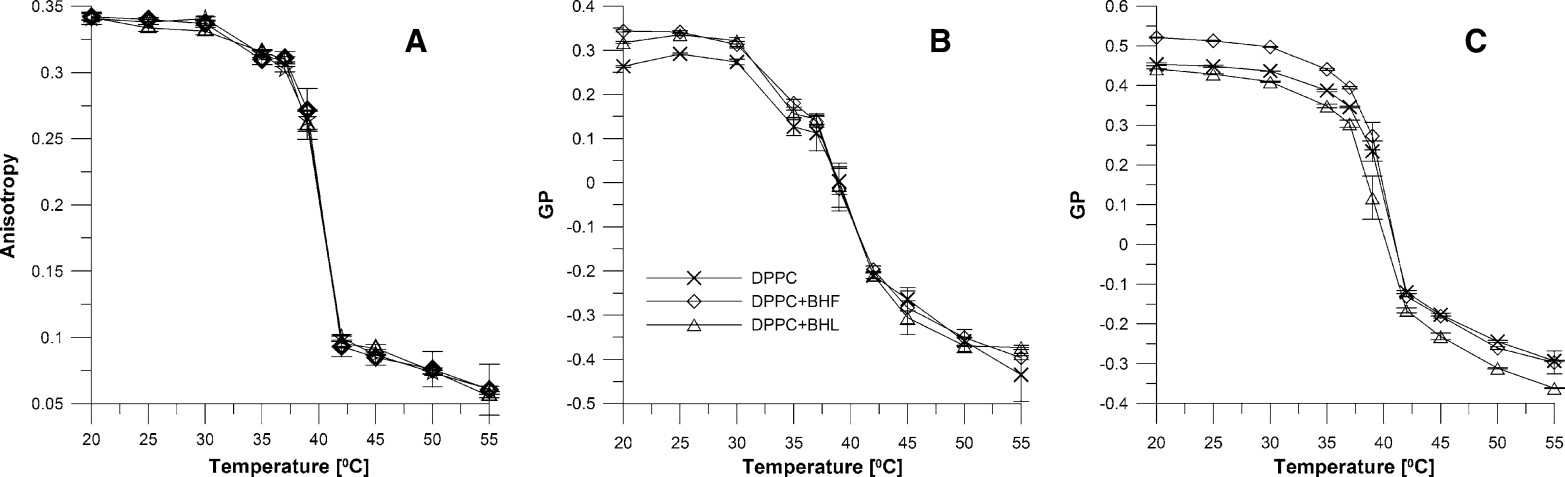
Values of fluorescence anisotropy (**a**) and values of generalized polarization (GP) of Prodan (**b**) and Laurdan (**c**) in SUVs formed from DPPC in absence and presence of extracts (0.05 mg/ml) as a function of temperature. *Triangle* BHL; *diamond* BHF; cross DPPC

Temperature change of the main phase transition induced by BHL and BHF was also examined with the fluorimetric method at 0.05 mg/ml extract concentration. The dependence of DPH probe anisotropy on temperature for DPPC liposome membranes is presented in Fig. 3a. Using the DPH probe, we assessed how fluidity of the model membrane modified with polyphenols in the extracts changes with temperature. In the area of hydrocarbon chains, no effect of the extracts was observed in the range of the main phase transition of DPPC. We did not observe any changes in anisotropy with temperature in the presence of BHL and BHF, which indicates that the extract compounds interact with the membrane only at the surface.

By using two fluorescent probes, Prodan and Laurdan, the packing order of the hydrophilic region of lipid membranes formed from DPPC in correlation to temperature was investigated. The GP of Prodan in SUVs formed from DPPC is presented in Fig. 3b as a function of temperature. The presence of extracts at a concentration of 0.05 mg/ml does not result in substantial changes to the temperature of the main phase transition of DPPC. The BHL and BHF extracts slightly increased the value of GP in the gel phase of DPPC but slightly decreased the value of GP in the liquid phase. Similar effects were observed for Laurdan (Fig. 3c). A significant increase in GP was observed in the presence of BHF, but only in the gel state. However, a decreased liquidity of the gel and liquid-crystalline phase of DPPC were observed in the presence of the BHL extract.

### Capacitance of Black Lipid Membranes

**Fig. 4:**
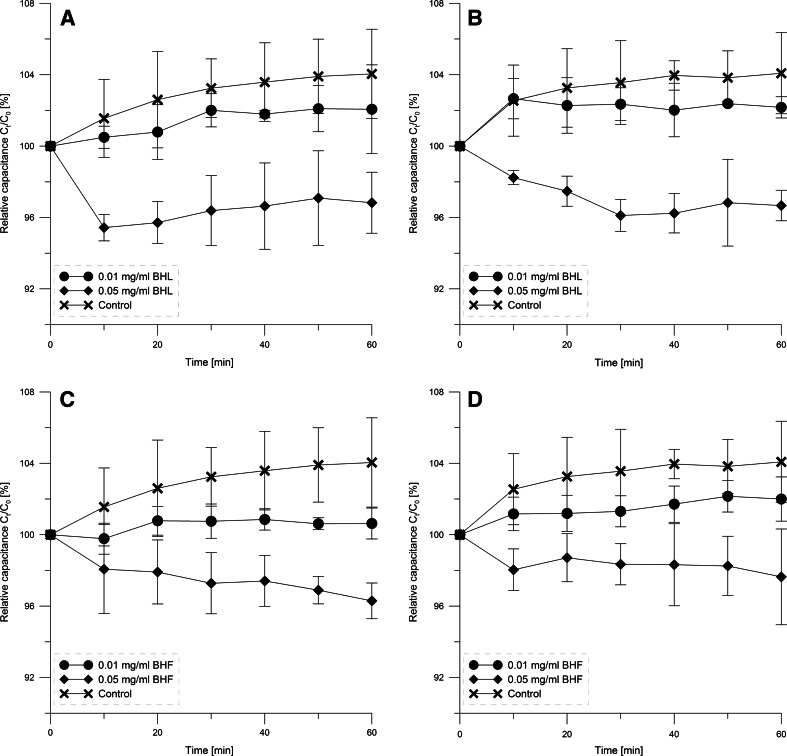
The dependence of relative capacitance (*C*_0_/*C*_t_) of black lipid membranes on time. **a** BLM_E_ in the presence of BHL extract, **b** BLM_EPC_ in the presence of BHL extract, **c** BLM_E_ in the presence of BHF extract, **d** BLM_EPC_ in the presence of BHF extract

We examined the effect of extracts of blue honeysuckle on electric capacity of BLM formed from EPC (BLM_EPC_) and RBCL (BLM_E_). After adding the extract to an electrolyte bathing a BLM, we observed a disturbance in the electrolyte–lipid bilayer equilibrium. Representative curves for relative change in capacity, BLM_E_ and BLM_EPC_, as a function of time in the presence of extracts, are given in Fig. 4.

The results obtained indicate that the presence of extracts in the lipid bilayer medium decreases the bilayer capacitance (Fig. 4), depending on the concentration of extract. At a higher concentration of 0.05 mg/ml for membranes formed from various lipids (BLM_E_ and BLM_EPC_), a significant decrease in specific capacity in relation to unmodified membranes was observed. This fall indicates that the substances contained in the extracts interact with the hydrophilic part of the lipid bilayer. In accordance with formula (2), the decrease in specific capacity of BLM, and therefore a larger increment in Δ*S* than Δ*C*, may be caused by changes in the thickness of the membrane (*d*) and/or changes in its electric permittivity. The extracts used, due to their hydrophilic character, interact with the surface of the membrane and modify its stability, and the character of the modification depends on the type of lipids used to create the BLM. For BLM_EPC_, the extracts caused a small destabilization of the lipid bilayer and for BLM_E_ an improvement in its stability. However, all the observed changes in capacity resulting from the effects of extracts on membranes are much smaller than the accepted criterion of stability (<10 pF/min), so their interaction with the membranes does not destructively affect the properties and structure of the lipid bilayer.

### Hydration of Lipid Membranes

**Fig. 5:**
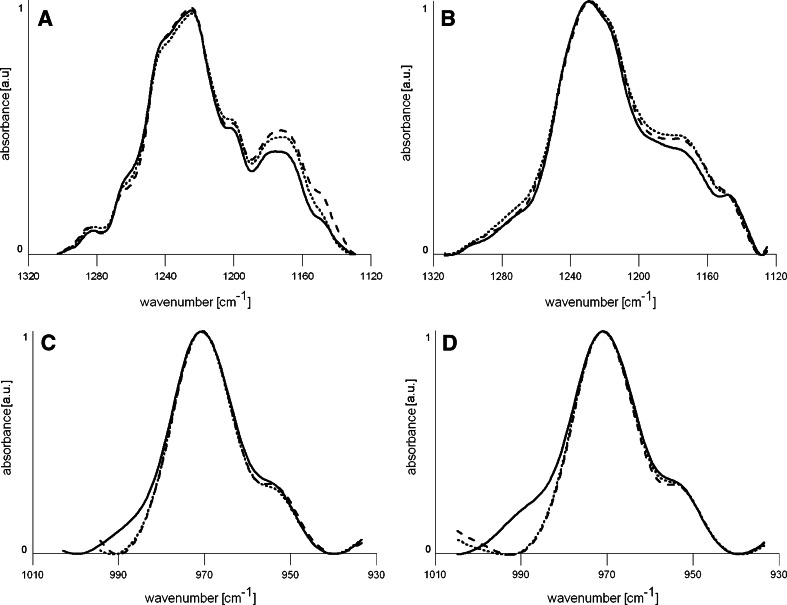
Phosphate band of DPPC (**a**), and EPC (**b**), choline band of DPPC (**c**), and EPC (**d**). *Solid line* control liposomes, *dashed line* liposomes with BHF extract, *dotted line* liposomes with BHL extract. Extract concentration was 0.05 mg/ml

At the used concentrations of the tested extracts, no changes in the hydrophobic part of the lipid membrane were observed in any spectra. The BHL and BHF extracts did not cause meaningful changes in the polar part of the membrane, while causing slight changes in the phosphate and choline bands of the spectra, which indicates a weak interaction of these compounds with the surface part of the membrane. Figure 5 shows the phosphate (a, b) and choline (c, d) bands of spectra for DPPC and EPC suspensions in the presence of BHL and BHF at 0.05 mg/ml. Figure 5a shows the phosphate band for DPPC liposomes. At room temperature, the membrane is in the gel phase. The maximum band is achieved for the wavenumber of 1225. The presence of the extracts slightly changed the relative intensity of the various parts of the band. The leaf extract increased intensity of the component of the wavenumber 1245, and the fruit extract reduced this wavenumber slightly. Figure 5b shows the phosphate band of EPC liposomes. Their membranes at room temperature are in the liquid-crystalline phase. There are no differences between the control probe and EPC liposomes formed in the presence of fruit extracts. Clearly different spectra are obtained in the presence of BHL. The whole band is shifted toward lower wavenumbers. These changes indicate a significant increase in the degree of hydration of phosphate groups of the membranes due to the presence of the BHL extract. Figure 5c, d shows the choline band of liposomes. The choline band of liposomes formed from DPPC (Fig. 5d) indicates a marked reduction in the band width at half maximum in the presence of tested extracts. The changes observed with EPC membranes are much less conspicuous.

### Antioxidant Activity of Extracts

The examination of the antioxidant activity of BHL and BHF extracts was made on erythrocyte membranes. Oxidation extent was determined in relation to membrane lipids and assayed fluorimetrically on the basis of kinetics of DPH-PA fluorescence quenching by free radicals induced by UVC radiation and the AAPH inducer.

Based on the kinetics of the oxidation curves obtained for various concentrations of both extracts and the antioxidant Trolox^®^, the concentration responsible for 50 % oxidation inhibition of erythrocyte membrane (IC_50_) was found. The IC_50_ ± SD [µg/ml] values obtained for erythrocyte membranes treated with BHL, BHF, and Trolox^®^ were as follows: for oxidation induced by UVC irradiation, BHL 23.2 ± 2.6, BHF 38.9 ± 1.8, and Trolox^®^ 14.6 ± 1.3; and for oxidation induced with AAPH: BHL 4.5 ± 0.65, BHF 4.0 ± 0.80, and Trolox^®^ 3.9 ± 0.3.

The results indicate that the extracts protect the lipids contained in red blood cell membranes against free radicals induced by UVC radiation and the AAPH inducer. The protection of membrane lipids demonstrated is comparable to the protection provided by Trolox^®^ in the case of AAPH, and smaller than the protection which Trolox^®^ provides against free radicals induced with UVC radiation. The results obtained show that BHL protects the erythrocyte membrane against UVC-induced oxidation better than BHF but worse than Trolox^®^. For protecting membranes against AAPH-induced oxidation, the BHL and BHF extracts were as good as Trolox^®^. Such results suggest that the reaction with free radicals depends on the oxidation inducer. In the case of photo-oxidation, the kinetics of the inhibition process is slower, whereas the kinetics of the oxidation process induced by AAPH is much faster, and the inhibition more efficient in the presence of extracts. These results suggest that polyphenols contained in the BHL and BHF extracts show very good antioxidant properties and have greater affinity to free radicals formed in oxidation induced by AAPH than UVC radiation.

## Discussion

The main polyphenolic components of the BHF extract are derivatives of cyanidin (approximately 84 % of all the polyphenols), and in particular cyanidin-3-glucoside, whose content in the extract is approx. 72.9 % of all the polyphenols. Cyanidins and their glycosides were reported to have a number of beneficial properties for human health that are related to their ability to scavenge free radicals, including reactive oxygen species (Chen et al. 2006; Rop et al. 2011). A different polyphenol composition is observed in the BHL extract, where phenolic acid derivatives and quercetin dominate, together accounting for approx. 98 % of total polyphenol content of the extract. Many works on chlorogenic acid derivatives and quercetin have assigned to these substances a range of healing and antioxidant activities (e.g., Pawlikowska-Pawlęga et al. 2003; Zhinan and Zhengxiang 2008). Very good antioxidant properties of chlorogenic acid and quercetin-3-O-glucoside in relation to biological membranes were observed in our earlier studies (Cyboran et al. 2011; Bonarska-Kujawa et al. 2011b). In the present study, various membrane models were used, from the simple one-lipid DPPC membrane, through more complicated membranes of liposomes formed from a mixture of EPC and lipids extracted from erythrocytes, up to the most complicated biological membrane (the erythrocyte membrane) which contains, apart from lipids, proteins, and sugars and their derivatives. In the present study, the following methods were used: fluorimetric, electric, calorimetric, and spectrophotometric (including infrared).

The results of this biophysical research have shown that polyphenolic compounds contained in BHL and BHF extracts induce changes in biological and lipid membranes, especially interacting with the lipids of biological membranes.

The investigations of amphiphilicity, aimed at specifying the affinity of the extracts to the organic (octanol) and aqueous (phosphate buffer) phase, have shown that compounds present in the extracts are of hydrophilic nature, and the components of honeysuckle fruit extract are more hydrophilic than those present in the leaf extract. Hemolytic tests showed that polyphenols contained in the extracts do not induce hemolysis and therefore do not exert lytic action on red blood cells in the range of concentrations studied. This result indicates that compounds present in the tested extracts do not penetrate deep into the hydrophobic part of the membrane. Hemolysis caused by various lytic compounds, in particular those of amphiphilic character, occurs when such substances penetrate into the alkyl chains region of the membrane, weakening the interaction between membrane components, and thus facilitating water transport into the cell interior with resulting structural damage. The lack of hemolysis for plant extracts was also confirmed in Bors et al. (2012), and Włoch et al. (2013). One can therefore assume that polyphenols from the extracts are present in the hydrophilic part of the membrane and do not embed deep into the hydrophobic membrane area.

The study of osmotic resistance confirmed the results of the hemolytic research, showing that, as a result of the combined action of the substances contained in the extracts, the osmotic resistance becomes slightly greater. The 50 % hemolysis of modified erythrocytes with lower concentrations of NaCl hypotonic solutions seems to be connected with decreased permeability to water molecules.

As evidenced by the microscopic investigation, both extracts induced changes in erythrocyte shape from the normal discoid to echinocytic form. Both extracts are responsible for creation of varied forms of echinocytes, mainly echinocytes (BHF) or echinocytes and spheroechinocytes (BHL). It can thus be assumed, according to the bilayer couple hypothesis (Sheetz and Singer 1974), that the extracts concentrate mainly in the outer monolayer of erythrocyte membrane when inducing various forms of echinocytes and practically do not permeate into the inner monolayer of the membrane. The presence of the extract compounds in the outer monolayer of erythrocyte membrane may cause its sealing, which also prevents hemolysis. Accumulation of polyphenolic compounds in the hydrophilic part of the membrane causes an increase in its area, which in turn leads to the formation of projections and the echinocytic shape of erythrocytes. This process is accompanied by a change in membrane hydration, which is implied by results of this FTIR investigation and those by Pawlikowska-Pawlęga et al. (2003) and Zhang et al. (2006). Based on the results of the microscopic examination, one can infer that the extract polyphenol molecules cause a lateral expansion of the erythrocyte membrane outer moiety, which results in cell shape change.

These results, obtained for erythrocytes, were confirmed on isolated erythrocyte membranes and model lipid membranes by the fluorescence, FTIR and DSC experiments.

The steady-state fluorimetry results of experiments performed with the DPH fluorescent probe that anchors in the hydrophobic part of the membrane, for all of the studied membranes, attest to small changes in membrane anisotropy. The lack of essential changes in membrane fluidity with respect to the control in this hydrophobic region inspired the authors to conduct studies using the Laurdan and Prodan probes, which can monitor changes in the hydrophilic region of the membrane. Laurdan completely partitions into the membrane, while Prodan partitions between water and polar head groups of the membrane and strongly depends on the state of the membrane phase. The fluorophore of Laurdan probe is located in the phospholipid glycerol backbone and is closely related to the dynamic and free movement of water molecules surrounding the Laurdan chromophore (Parasassi et al. 1998). Decreased GP values in the tested membranes seemed to indicate decreased packing arrangement of the membrane and lipid-water interface hydration. A significant decrease in generalized polarization of Laurdan probe was observed with the BHL and BHF extracts at a concentration of 0.05 mg/ml in all the membranes tested. Such changes in GP of Laurdan probe may indicate at polyphenol molecules binding to the membrane surface and expelling water molecules into the polar head region which leads to growth in membrane hydration and change of the membrane packing arrangement. Using the Prodan probe, slight changes were observed in GP, in the presence of extracts, in the liquid-crystalline phase and in the gel phase of DPPC liposomes.

The DSC results showed a lack of change in the phase transition temperature of DPPC membranes, indicating that there were no changes in the hydrophobic part of the lipid bilayer, which demonstrates the absence of extract polyphenolics in that region. In addition, they do not cause changes in the fundamental structure and organization of the phospholipid bilayer. Increased concentration of the extracts caused only a reduction in the DPPC pre-transition, which may suggest that tested compounds cause only small changes in the polar part of the membrane.

The investigation of the effect of the extracts on BLM capacity showed that the extracts cause a decline in the electric capacity of BLM, which proves their effect on the hydrophilic membrane area (Movileanu et al. 2000). The observed decrease in specific capacity of BLM may be caused by changes in thickness of the membrane and/or changes in its permeability. Membrane thickness (*d*) may change slightly due to penetration of molecules of modifying substances into the inside of the membrane or adsorption on its surface. The adsorption on the surface of the membrane probably leads to changes in the membrane electrical permeability. Research on BLM capacity showed that the extracts tested slightly stabilize the black lipid membrane created from lipids isolated from erythrocyte membranes.

Research on IR spectra indicates changes in the degree of hydration in the hydrophilic region of liposomes created from DPPC and EPC, in the phosphate and choline band under the action of extracts. The presence of the extracts does not cause spectral shifts in the hydrophobic area of the hydrocarbon chains but causes shifts in the spectrum, corresponding to vibrations of the phosphate and choline group. These results indicate binding of extract ingredients in the hydrophilic region of the polar heads of the lipid membrane. As suggested by Pawlikowska-Pawlęga et al. (2013), as a result of the effects of polyphenols on lipid membranes, it is likely that hydrogen bonds form between the hydroxyl groups of polyphenols and the lipid polar groups.

The lack of essential changes with respect to the control in the hydrophobic region and significant changes in the hydrophilic region could mean that the components of the extracts interact with the surface of the membrane. The BHL and BHF extracts exhibited high antioxidant activity toward reactive forms of oxygen that developed as a result of membrane photo-oxidation induced by UVC radiation and by the AAPH inducer and were also tested by the fluorimetric method. The presence of polyphenols in the hydrophilic region of the erythrocyte membrane suggests a possible mechanism of their antioxidant action. On the one hand, being present on the membrane surface and in the medium, they reduce the concentration of free radicals by scavenging them, and on the other hand, they restrict diffusion of the radicals into the membrane.

## Conclusion

The polyphenolic compounds contained in the BHL and BHF extracts do not disrupt the structure of the biological membrane and do not penetrate deep into the hydrophobic region of erythrocyte and lipid membranes, as is evident from the hemolytic, microscopic, calorimetric, electric, and FTIR investigations. They become located in the hydrophilic region of the membrane and stabilize the membrane structure by changing its hydration. The results obtained indicate that blue honeysuckle leaf and fruit extracts effectively protect the cell membrane against oxidation. On the basis of the results, one can infer that the protective action of the extract components with respect to biological membranes depends on the number of polyphenol molecules adsorbed to the membrane surface. The polyphenols contained in the extracts reduce the concentration of free radicals, constituting a specific protective barrier which hinders infusion of free radicals into the membrane.

Thus, the results of the research encourage consumption of food products with the ingredient of blue honeysuckle fruit or leaf, which can scavenge free radicals and boost our immunity to many diseases related to oxidation stress.

## Abbreviations

AAPH: 2,2′-azobis (2-methylpropionamidine) dihydrochloride
DPH: 1,6-Diphenyl-1,3,5-hexatriene
DPH-PA: (1,6-Diphenyl-1,3,5-hexatriene) propionic acid
DPPC: 1,2-Dipalmitoyl-sn-glycero-3-phosphatidylcholine
DSC: Differential scanning calorimetry
EPC: Egg yolk lecithin
GP: Generalized polarization
Laurdan: 6-Dodecanoyl-2-dimethylaminonaphthalene
Prodan: 6-Propionyl-2-dimethylaminonaphthalene
BLM: Black lipid membranes
RBCL: Red blood cell lipids
MLV: Multilamellar vesicles
SUV: Small unilamellar vesicles
BHL: Blue honeysuckle leaf extract
BHF: Blue honeysuckle fruit extract
